# Prioritizing the Needs of Caregivers of Older Adults to Support Their Help-Seeking Process as a First Step to Developing an eHealth Tool: The Technique for Research of Information by Animation of a Group of Experts (TRIAGE) Method

**DOI:** 10.2196/12271

**Published:** 2019-05-23

**Authors:** Karine Latulippe, Mélanie Tremblay, Valérie Poulin, Véronique Provencher, Anik MC Giguere, Andrée Sévigny, Véronique Dubé, Sophie Éthier, Manon Guay, Maude Carignan, Dominique Giroux

**Affiliations:** 1 Department of Teaching and Learning Studies Laval University Quebec, QC Canada; 2 Université du Québec in Trois-Rivières Trois-Rivières, QC Canada; 3 Interdisciplinary Center for Research in Rehabilitation and Social Integration Quebec, QC Canada; 4 School of Rehabilitation University of Sherbrooke Sherbrooke, QC Canada; 5 Research Centre on Aging Centre intégré universitaire de santé et de services sociaux de l’Estrie-Centre hospitalier universitaire de Sherbrooke Sherbrooke, QC Canada; 6 Department of Family Medicine and Emergency Medicine Laval University Quebec, QC Canada; 7 Center of Excellence on Aging Quebec Quebec, QC Canada; 8 School of Social Work and Criminology Laval University Quebec, QC Canada; 9 Faculty of Nursing University of Montreal Montreal, QC Canada; 10 Research Center of the University Hospital of Montreal Montreal, QC Canada; 11 Department of Rehabilitation Laval University Quebec, QC Canada

**Keywords:** caregivers, aged, help seeking behavior, community-based participatory research, eHealth, telemedicine

## Abstract

**Background:**

Caregivers of functionally dependent older persons sometimes seek formal services to support their relatives. However, this process of help-seeking is complex.

**Objective:**

The overall aim of the study was to use a co-design approach to develop an electronic health (eHealth) tool to support caregivers in their process of help-seeking. This study presents the first step of the design phase, which aimed to prioritize the user needs to be considered during the development of an eHealth tool.

**Methods:**

A total of 3 groups of caregivers, community workers, and health and social service professionals participated in either a co-design session (1 or 2) or an advisory committee in 2 rural areas and 1 urban area. The needs identified in the academic literature and during a previous study were sorted (Technique for Research of Information by Animation of a Group of Experts [TRIAGE] method) by the participants (referred to in this study as co-designers) to obtain a consensus on those to be prioritized. Needs identified, grouped, and removed were ranked and compared.

**Results:**

Of the initial list of 32 needs, 12 were modified or merged, 3 added, and 7 deleted as the co-designers felt that the needs were poorly formulated, redundant, irrelevant, or impossible to meet. In the end, 19 needs were identified for the design of the eHealth tool.

**Conclusions:**

Many of the identified needs are informational (eg, having access to up-to-date information) and are probably met by existing tools. However, many others are emotional (eg, being encouraged to use the services) and offer an interesting challenge to eHealth tool development.

**International Registered Report Identifier (IRRID):**

RR2-10.2196/11634

## Introduction

### Background

In Quebec, an estimated 296,000 men and 402,700 women are caring for their parents or in-laws, and approximately 80,200 men and as many women take care of their life partners [1]. About 8.1 millions of Canadians aged 15 years and above reported providing care to a family member or friend with a chronic condition, a disability, or age-related problems in the 12 months preceding a 2012 survey [2]. Care provision included transportation, meal preparation, bathing and clothing, and help with medical treatments. It can be assumed that this number has since increased. Quebec, like many other parts of the world, has an aging population [3]. The increase in the proportion of people aged 65 years or above will continue in Canada (including Quebec) in the coming years. This group will represent between 23% and 25% of the population in 2036 and between 24% and 28% in 2061 compared with 14% in 2009 [4]. The aging of the population is leading to an increase in the demand for support for older persons and, consequently, a rise in the number of caregivers [1].

Furthermore, one of the difficulties encountered by caregivers is the search for formal services to assist them in their role (services for themselves or for the elders they support) [5,6]. Increasingly, caregivers are turning to the internet to begin their search. Electronic health (eHealth) can support caregivers in this process [7-11]. Eysenbach defines eHealth as an emerging field at the intersection of medical information technology, public health and business, and referral to health services and information delivered or enhanced through the internet and related technologies [12]. It is from this perspective that this study emerged. Funded by the Quebec Ministry of the Family, this study aimed to develop an eHealth tool to support the process of help-seeking by caregivers of elderly people [13]. The development of this tool is intended to be inclusive, that is, the research team wanted all caregivers, regardless of their technological skills, level of education, or numerical literacy, to be able to use the tool efficiently. Furthermore, one of the promising interventions to achieve this is to develop the tool with future users; in this case, caregivers and potential health and social service professionals (HSSPs) and community workers [14]. Therefore, the chosen approach was co-design. Co-design refers to the creativity of designers and people not trained in design, working together in the design development process [15]. Thus, caregivers acting as designers can intervene directly in their future eHealth tool and draw upon their knowledge to develop technologies that respect their needs and their ways of doing things [13,16]. In this study, co-design started at the first step of the design phase to define the problem and understand the needs of caregivers [17]. This study specifically presents this phase of the study.

### Needs of Caregivers

According to Amieva et al, caregivers’ expectations and needs are principally twofold: first, the ready availability of information on the disease and, second, the acquisition of skills to optimize the help given to the patient on a daily basis [18]. Dunbrack, for his part, pointed out that the following needs are the most common: pain relief, grief support, respite, information about caregiving and illness, knowledge of how to deal with professionals and volunteers (knowing who does what), help with answering legal and financial questions, and emotional and spiritual support [19]. He also added that it is important to recognize changing information needs so that both the caregiver and the health care team can anticipate and plan for such changing needs [19]. Caregivers mentioned that they preferred oral communication with information in a form that they could refer to repeatedly to assimilate it more effectively (eg, a booklet, a book, a website, or a video) and return to over time to refresh their memory or fill in a blank [19]. The need for well-coordinated postdiagnostic support, greater continuity of care with regard to the personnel involved, and enhanced access to nonpharmacological interventions to support identity and social engagement was also found to be important for caregivers [20]. Another study found that family ties and affection make it difficult for those accompanying a loved one to identify themselves as caregivers. Lack of support or information about available supports, insufficient time and energy, a focus on the needs of the accompanied person, and inadequate cooperation with professionals are also obstacles preventing family caregivers from becoming aware of their own needs and expressing them [21]. It emerges from this study that isolation is both a key factor and the main consequence of this lack of awareness. Finally, on the basis of a systematic review of the literature, Plöthner and his team have identified needs that include work-life balance, respite, the importance of trusting service providers, low service costs, obtaining information on existing services, and pathologies and symptom management, among others [22].

Nevertheless, although the needs of caregivers are known and services to meet them exist, nonuse of services persists. Earlier studies have identified the reasons caregivers fail to use formal services, including service-related factors (knowledge of available services, multiplication of procedures, home care, transportation, cost, and reliance on organizations), relational factors (feelings of guilt, insecurity, rejection by the elder, and isolation), information factors (network knowledge, current and centralized information, and proactivity of stakeholders), experiential factors (previous experience with organizations and the extent of the burden), and personal factors (the caregiver’s ability, denial, and self-identification) [23,24]. Bieber et al identified perceptions of useful services, misunderstood by health care professionals in terms of the level of burden experienced, the competence of the informal caregivers in providing care, little knowledge of available services, and difficulties in obtaining information about services owing to the complicated service system as all constituting barriers to the use of services [25]. On the basis of these earlier studies, this study aimed to prioritize the needs of caregivers of elderly people that can be met by an eHealth tool to support the process of help-seeking.

## Methods

### Research Design

**Figure 1:**
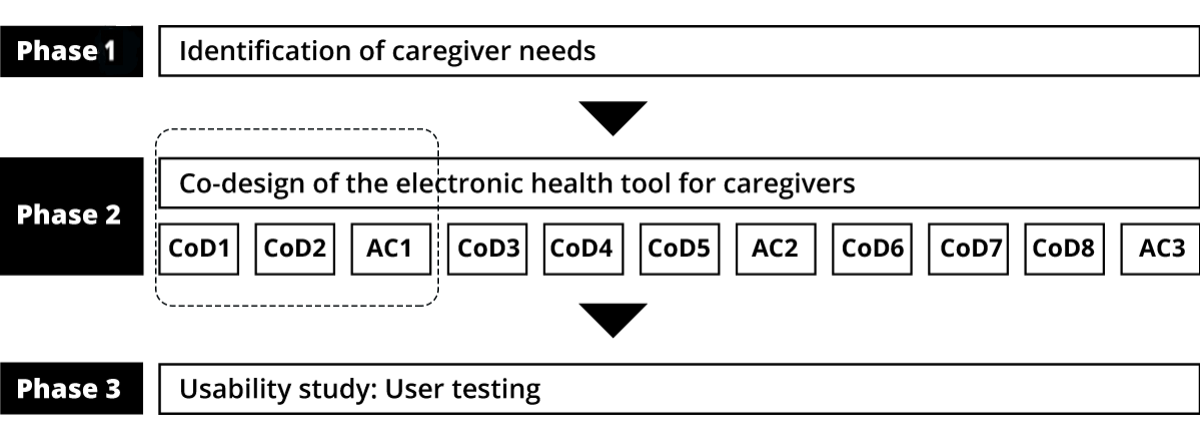
Design phase of the entire project and steps involved in this publication (in a box). CoD: co-design session; eHealth: electronic health.

This study was part of a broader participatory study using a 3-phase co-design approach: (1) identifying the needs of caregivers in terms of tools to support their help-seeking process, (2) developing a tool for caregivers corresponding to the needs expressed, and (3) testing the usability of the tool (see Latulippe et al [13] for more details). This study presents the first step of the design phase (box of Phase 2—Figure 1). Furthermore, 3 groups of caregivers, community workers, and HSSPs participated in either a co-design session (CoD1 or CoD2) or an advisory committee (AC).

The needs of caregivers have been examined in previous studies [22]. To avoid repeating these studies but rather build on them and deepen the reflection, we compiled a list of these needs. The needs identified in the academic literature and during a previous study [23] (see Multimedia Appendix 1) were sorted by the co-designers using a Technique for Research of Information by Animation of a Group of Experts (TRIAGE) method to obtain a consensus on those to be prioritized [26]. TRIAGE is a dynamic technique of information retrieval and, in some cases, decision making by a group of experts. First, 32 needs were presented, each written on a paper on the wall. By mutual agreement, co-designers had to choose, for each need, whether they place it in the basket (need to keep), in the trash (need rejected), or the refrigerator (need that did not reach consensus or that the co-designers could not choose). The co-designers could reformulate the needs, group them together if they considered them equivalent, or add some.

Next, by subgroup (caregivers together, community workers together, and HSSPs together), co-designers of the first CoD had to prioritize the needs identified in the basket, from most important to least important. We chose to make homogenous subgroups as we feared that caregivers would feel less comfortable taking a stand with workers. As the ways of prioritizing were very different from 1 group to another and made the analysis and choice of needs difficult, another technique was used for the second CoD. For the latter, after using the TRIAGE method (as for the first group), a comparison of the responses with group 1 was discussed in a plenary session, to understand the different reasoning underlying the choices. Then, each co-designer had to affix stickers (a maximum of 10) to the needs retained in the basket, which seemed a priority to him or her. Finally, the research team presented the results of Co-design 1 and 2 in a tabular form to the AC, highlighting points of convergence and divergence. The purpose of the AC was to decide on the needs to be retained for the development of the eHealth tool. A group discussion helped to achieve this goal.

### The Research Sites

The first 2 CoDs were held in cities in predominantly rural areas, namely Gaspésie (Grande-Vallée) and Côte-Nord (Baie-Comeau). The AC was held in Quebec City and included co-designers from the Capitale-Nationale and Chaudière-Appalache regions.

### Recruitment

A purposive sampling strategy was employed. For the HSSPs, direct contact was made with management of senior services. For community workers, a direct approach was employed after researching community organizations for caregivers in the targeted territory on the Web. Community workers and HSSPs willing to recruit caregivers to participate in the study, through a direct approach, were solicited (see Latulippe et al [13] for more details).

### Analysis

After each CoD, the research team was debriefed to elicit their first impressions (eg, any surprising exchanges). Identified, regrouped, and withdrawn needs were classified with Excel (Microsoft) by 1 member of the research team and then validated with the rest of the team. The needs retained in the 2 groups, as well as the prioritization, were compared. The AC decided on the needs to be retained by group consensus.

## Results

### Sociodemographic Data

The group in the first CoD included 3 caregivers, 2 community workers, and 2 HSSPs (a total of 7 co-designers). The group consisted solely of women aged 37 to 66 years. The group in the second CoD included 4 caregivers, 1 community worker, and 1 health and social services worker (a total of 6 co-designers). This latter group comprised co-designers aged 41 to 77 years, one of whom was a man. The AC consisted of 1 caregiver, 2 community workers, 2 HSSPs, and 3 researchers who collaborated on the project (8 co-designers in all). Table 1 presents the sociodemographic data of all the co-designers (CoD1, CoD2, and AC) who contributed to the identification of the needs, with the exception of the researchers.

**Table 1:** Sociodemographic data of all co-designers.

Sociodemographic data		Caregivers (n=8)		Community workers (n=5)		Health and social service professional (n=5)	
**Sex (n)**							
	Women		7		4		5
	Men		1		1		0
Age (years), range (mean)		42 to 77 (59.4)		25 to 66 (47.8)		33 to 53 (42.6)	
**Education level (n)**							
	Elementary school		1		0		0
	High school		2		1		0
	College		1		2		1
	University		4		2		4
Age of the relative (years), range (mean)		61 to 94 (73.4)		—^a^		—	
**Diagnosis of the relative (n)**							
	Pick's disease		1		—		—
	Autonomy loss		2		—		—
	Intellectual disability		1		—		—
	Muscular dystrophy		1		—		—
	Stroke		1		—		—
	Mental health disease		1		—		—
	Cancer		1		—		—
**Relationship to the person they provide care for (n)**							
	Children		3		—		—
	Sibling		2		—		—
	Spouse		2		—		—
	Friend		1		—		—
**Number of years to be a caregiver (years), range (mean)**							
	Between 1 and 60 years		20.3		—		—

^a^Not applicable as the notions of age, diagnosis, nature of the relationship with the relative and the number of years to be a caregiver do not apply to health and social service professional and community workers.

### Technique for Research of Information by Animation of a Group of Experts and Prioritization of Co-Design Session

**Figure 2:**
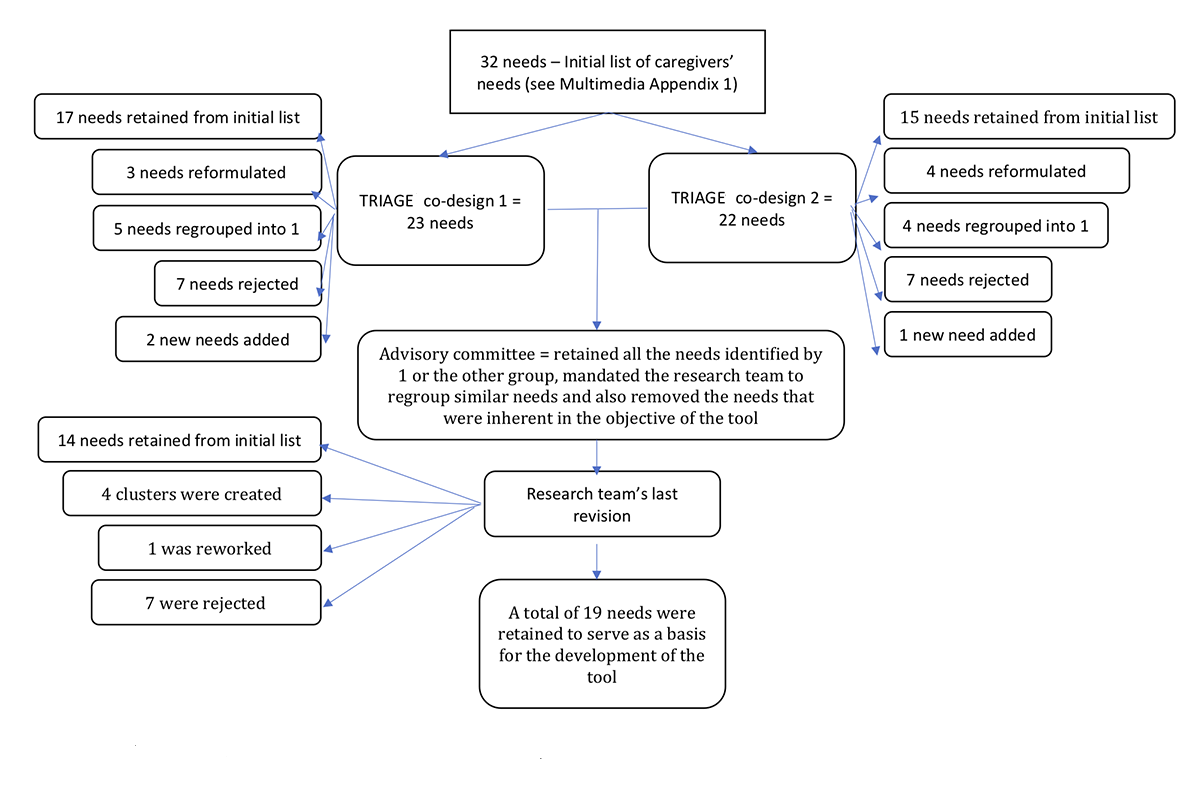
Diagram of the needs identification process used as the basis for the electronic health tool. TRIAGE: Technique for Research of Information by Animation of a Group of Experts.

The entire process is shown in Figure 2, and the details of the choices made are shown in Multimedia Appendix 2. The first co-design group retained 17 initial needs. Co-designers reformulated 3 and regrouped 5 needs into one. They rejected 7 and added 2 for a total of 23 needs to consider for the prioritization exercise.

Caregivers retained all the needs in the prioritization exercise. They put them in order of importance. The HSSP retained the needs in the prioritization exercise and prioritized 2 needs that had been rejected in TRIAGE (having access to a network of people who know the resources and having access to a service suitable for older persons). The group of community workers chose all 23 needs in their prioritization exercise and put 1 need in their prioritization section that had been rejected in TRIAGE (having access to a service offer suitable for elders), which they classified into 12 subgroups.

The second co-design group selected 15 initial needs (without having seen the results of the first co-design), reformulated 4, grouped 4 into 1 and 2 into 1, rejected 7, and added 1 need. Co-designers from this second group proposed 22 needs to be considered in the tool. At our request, they then prioritized 10 needs.

Finally, the AC decided to retain all the needs identified by the groups as important (classified in the basket during the TRIAGE exercise). The committee mandated the research team to regroup similar needs and also remove those that were inherent to the objective of the tool (eg, finding resources) or beyond the limits of what the tool can do (eg, offering coordinated services). The result of the prioritization (10 more priority needs) at Co-design 2 was, therefore, not considered more than the prioritization of Co-design 1. From the initial needs, 14 were retained, 4 clusters were created, 1 was reworded, and 7 were rejected. Therefore, a total of 19 needs were retained to serve as the basis for the development of the tool (Textbox 1).

**Needs retained to serve as the basis for the development of the tool.**

1. Having access to up-to-date information, anytime, anywhere
2. Having access to educational interventions
3. Having access to a service offer suitable for elders
4. Having access to a keyword search
5. Being able to add training workshops, resources, and activities
6. Knowing the service offer (costs, transportation, home-based care, eligibility criteria, and proximity)
7. Asking questions
8. Receiving information regularly
9. Having access to concise and simple tools
10. Having a choice of language
11. Being reassured about resources
12. Being able to access and keep information easily
13. Recognizing the needs
14. Recognizing themselves as caregivers
15. Being comfortable using the service
16. Being able to connect with people experiencing the same situation
17. Being encouraged to request help before reaching a state of exhaustion
18. Being encouraged to use the services
19. Being guided in the help-seeking process

## Discussion

### Principal Findings

In summary, the results obtained are as follows: of the initial list of 32 needs, 12 were modified or added, as the needs were poorly formulated, redundant, irrelevant, or impossible to meet. In the end, 19 needs were identified for the design of the eHealth tool. There were no identified needs that surprised the research team as the initial list of needs came from previous studies and was, therefore, well justified. The 3 additions made by the co-designers (knowing about the proximity of services, being informed about staff stability, and being able to add training workshops, resources, and activities) are also consistent with the literature. Proximity or lack of proximity to services can be a barrier to using services as the extra time required to get to the resource signifies a greater expenditure of time and money [27]. Thus, it is important that the caregiver finds resources nearby or at least knows that transportation will be needed to get there. In addition, limited access to transportation, in rural areas among others, can make use of the service impossible [25]. The need to be informed about staff stability is not a surprise for people who have worked in the health or community network. A recent study by our team raised this issue as a factor in the nonuse of formal services [23]. However, this need, although considered significant, was not retained as organizations have little control over this aspect, which can vary greatly over time. The last need added, being able to add training workshops, of course, is a request from community stakeholders to promote their activity. Despite marketing efforts, some activities of community organizations do not attract enough participants. However, the need for information (eg, methods or strategies for managing physical and psychological care and access to care services) is well documented and could be met through conferences or workshops [28,29].

However, what surprised and even unsettled the research team was the rejection of the need being advised by a peer from the first group. The reasoning behind this rejection was that it is a need that should be met by the services and not via a Web tool. Several academic studies have documented the importance of support groups or the contribution that a peer may make in helping a caregiver [23,28,30]. This has led to the first epistemological issue: wanting co-designers to really share decision-making leads to a challenge in reaching agreement when experts have different perspectives (on the basis of theory, experience, and practice). This issue was faced by the Hendricks team, which also argued that this is inevitable when a real co-design approach is used [31]. When the second group chose to prioritize this need, this raised another issue related to the methodology: what should be done when there is a difference in the choices of co-design groups? Submitting the results of the first group to the second group at the end of the session and discussing the differences with them helped to further the discussion. The importance of the AC was highlighted in the face of these 2 issues. Here, the intervention of a third party (the AC comprised both caregivers and workers) made it possible to reach decisions and to continue the project without the research team having to take a stand in favor of one group or another.

### Other Reflections on the Method Used

It is possible that basing reflection on an existing list of needs may influence the choice of co-designers. However, as this list comes from previous academic studies with caregivers, this appears to be useful data. In addition, the co-designer can completely modify the list (remove needs, add, or group them) and, thus, update this list. In our opinion, the fact that 3 different groups took part in this reflection and that, for each of the groups, there were caregivers, community workers, and HSSPs, also makes it possible to meet the scientific criteria of credibility. Finally, the fact that co-designers added 2 needs (to Co-design 1) and 1 need (to Co-design 2) demonstrates that co-designers were not passive in this reflection. We believe that this method makes it possible to meet the objective (identifying the needs of future users) efficiently, using a co-design approach, to allow more time for the development of functionalities and content of the eHealth tool.

Furthermore, the entire project covers several regions, including both rural and urban areas, to provide a variety of perspectives and includes a total of 74 co-designers. However, this study is about needs identification, the first part of the co-design phase of this project. This was carried out in 2 regions classified as rural (Co-design 1 and 2) and a city (AC) of the same province with a relatively small sample (21 co-designers in total). The identified needs of caregivers living in urban or rural areas may differ [25]. Thus, we can question the transferability of the data obtained. However, the choice of the method of TRIAGE from a list on the basis of the scientific literature (and consequently according to different perspectives) reassures us as to the transferability of the needs chosen for the continuation of the phase of co-design on one hand but also for the utility that this can represent for the academic and clinical community.

Although we believe that the needs identified can be applied to both rural and urban areas, this method does highlight the specific features of each of the regions. For example, consider the Aboriginal community on the Côte-Nord and the issue of confidentiality, given the small size of the community and the proximity of people in Grande-Vallée. To allow several regions to share their uniqueness, the rest of the project took place in different regions, and it was possible for co-designers to discuss the particularities of their region.

### Limitations

Although this was not deliberate, the ethnicity of the co-designers was almost entirely Caucasian. Considering that culture greatly influences the perception of the role of a caregiver and the relationship with health services, it can be assumed that needs will be different for other communities [32]. Another limitation is that several caregivers had the dual role of being both community workers or HSSPs and caregivers. This may have influenced the choice of needs as knowledge of the health network before the role of a caregiver is a facilitator of resource use [23]. At the same time, it is also a reality. Caregivers necessarily take on several roles at once. Finally, the strategy for prioritizing needs was modified for the second group to limit the number of needs that would serve as the basis for developing the tool. To our knowledge, there are no recommendations as to the number of needs that can be used as the basis for a Web tool. At that moment, the fear was of not being able to meet all the stated needs. This change limited the ability to compare groups. Nonetheless, the AC decided to keep the initial needs that had been retained in the basket during the TRIAGE exercise, justifying their decision by the fact that if the need had been kept, it was considered important. In this sense, the prioritization exercise appears to be unnecessary.

### Conclusions

Using a co-design approach and the TRIAGE method, caregivers of functionally dependent older persons, community workers, and HSSPs identified 19 needs serving as the basis for designing an eHealth tool to support the help-seeking process. The important objective of having access to up-to-date information at any time and in any place, educational and adapted interventions, a keyword search tool, information on the formal services offered, and the possibility of asking questions, receiving information regularly, and retrieving information effortlessly (needs expressed by co-designers) can be achieved quite easily with an eHealth tool, as long as it is simple and concise and in the future user’s language. Nevertheless, in this study, the innovative challenge offered by the co-designers is that of also responding to more emotional needs, such as being reassured about service providers, recognizing one’s own needs and those of the elder, recognizing oneself in the role of a caregiver, being comfortable using formal services, being in contact with peers, being encouraged to seek help before symptoms of fatigue appear, and, finally, being guided through the process of seeking help through an eHealth tool. The next step of the project involves co-designers (caregivers, community workers, and HSSPs) being asked to choose and develop functional and content requirements that meet the selected needs and, therefore, respond to this challenge. It is likely that several existing tools (Web and apps) for caregivers meet some of the needs identified in this study, especially those of an informational nature. As the eHealth tool targeted by this project is intended to complement what already exists, the next step of the study will be to analyze, with the co-designers, the tools available to identify the needs already met versus the needs yet to be met.

## Appendix

Multimedia Appendix 1 Initial list of caregivers' needs.

Multimedia Appendix 2 Results of the Technique for Research of Information by Animation of a Group of Experts and decisions taken by the advisory committee.

## Abbreviations

AC: advisory committee
CoD: co-design session
eHealth: electronic health
HSSP: health and social service professional
TRIAGE: Technique for Research of Information by Animation of a Group of Experts
