# Supplementary material for: Prioritizing the Needs of Caregivers of Older Adults to Support Their Help-Seeking Process as a First Step to Developing an eHealth Tool: The Technique for Research of Information by Animation of a Group of Experts (TRIAGE) Method
Source: JMIR Aging. 2019 May 23;2(1):e12271. doi: 10.2196/12271 (PMC6716487; doi:10.2196/12271)
Supplement: Multimedia Appendix 1 [file aging_v2i1e12271_app1.pdf]

Multimedia Appendix 1: Initial list of caregivers' needs

1. Have access to educative interventions
2. Have access to a service offer corresponding to the elder profile
3. Have access to concise and simple tools
4. Have a choice of language
5. Have access to services anywhere
6. Have access to services at all times
7. Have access to a network of people who know the resources
8. Have access to a keyword search
9. Have services adapted to a varied schedule
10. Have up-to-date information
11. Have information grouped in one place
12. Have coordinated services
13. Have a service offer that anticipates future needs
14. Know the eligibility criteria of a service
15. Know the qualifications of the staff
16. Know the resources and services
17. Know the resources that offer home service
18. Know the resources that offer transportation
19. Know low-cost services
20. Be advised by a peer
21. Be encouraged asking help before exhaustion
22. Be encouraged using the services
23. Be guided in identifying needs
24. Be guided in the help-seeking process
25. Be reassured about resources
26. Ask questions
27. Be able to keep and retrieve information easily
28. Receive information regularly
29. Recognize the problem
30. Recognize themselves has caregivers
31. Feel less guilty about using services
32. Find resources
