# Supplementary material for: Prioritizing the Needs of Caregivers of Older Adults to Support Their Help-Seeking Process as a First Step to Developing an eHealth Tool: The Technique for Research of Information by Animation of a Group of Experts (TRIAGE) Method
Source: JMIR Aging. 2019 May 23;2(1):e12271. doi: 10.2196/12271 (PMC6716487; doi:10.2196/12271)
Supplement: Multimedia Appendix 2 [file aging_v2i1e12271_app2.pdf]

Multimedia Appendix 2. Results of the TRIAGE and decisions taken by the advisory committee.

| Initial needs | Co-design 1 | | | Co-design 2 | | | AC[a] and research team |
|---|---|---|---|---|---|---|---|
| | Basket | Trash | Adding | Basket | Trash | Adding | Decision |
| Having access to educative interventions | ✔ | — | — | ✔ | — | — | Kept |
| Having access to a service offer corresponding to the elderly person's profile | — | ✔ | — | ✔ | — | — | Kept |
| Having a service offer that anticipates future needs | — | ✔ | — | ✔ | — | — | Rejected because: The tool has no control over it |
| Having access to concise and simple tools | ✔ | — | — | ✔ | — | — | Kept |

| Initial needs | Co-design 1 | | | Co-design 2 | | | AC[a] and research team |
|---|---|---|---|---|---|---|---|
| | Basket | Trash | Adding | Basket | Trash | Adding | Decision |
| Having access to a network of people who know the resources | — | ✓ | — | Modified to: Developing a network of people who know the resources | — | — | Replaced by: Being able to connect with people experiencing the same situation |
| Being advised by a peer | — | ✓ | — | Modified to: Being able to connect with people experiencing the same situation | — | — | |
| Having access to a keyword search | ✓ | — | — | ✓ | — | — | Kept |
| Having access to information anywhere, anytime | ✓ | — | — | ✓ | — | — | Replaced by: Having access to up-to-date |

| Initial needs | Co-design 1 | | | Co-design 2 | | | AC[a] and research team |
|---|---|---|---|---|---|---|---|
| | Basket | Trash | Adding | Basket | Trash | Adding | Decision |
| Having up-to-date information | ✓ | — | — | ✓ | — | — | information, anytime, anywhere |
| Having access to services at all times | — | ✓ | — | ✓ | — | — | |
| Having information collected in one place | ✓ | — | — | ✓ | — | — | Rejected because: This is part of the tool. |
| Having services and courses adapted to a varied schedule | ✓ | — | — | ✓ | — | — | Rejected because: The tool has no control over it |
| Having a choice of language | ✓ | — | — | ✓ | — | — | Kept |

| Initial needs | Co-design 1 | | | Co-design 2 | | | AC[a] and research team |
|---|---|---|---|---|---|---|---|
| | Basket | Trash | Adding | Basket | Trash | Adding | Decision |
| Being encouraged to use the services | ✓ | — | — | — | ✓ | — | Kept |
| — | — | — | Knowing the proximity of services | — | — | — | Replaced by: Knowing about the service offer (costs, transportation, home-based care, eligibility criteria, and proximity) |
| Knowing the eligibility criteria of a service | Modified to: Knowing about the service offer (costs, transportation, home-based care, eligibility | — | — | ✓ | — | — | |
| Knowing about the resources and services | | — | — | — | ✓ | — | |
| Knowing about the resources that offer home service | | — | — | Modified to: Knowing about the service offer | — | — | |

| Initial needs | Co-design 1 | | | Co-design 2 | | | AC[a] and research team |
| --- | --- | --- | --- | --- | --- | --- | --- |
| | Basket | Trash | Adding | Basket | Trash | Adding | Decision |
| Knowing about the resources that offer transportation | criteria, and proximity) | — | — | (cost, transportation, home, and staff qualifications) | — | — | |
| Knowing about inexpensive services | | — | — | | — | — | |
| Knowing the qualifications of the staff | ✓ | — | — | | — | — | Rejected |
| Being encouraged to ask for help before reaching a state of exhaustion | ✓ | — | — | ✓ | — | — | Kept |

| Initial needs | Co-design 1 | | | Co-design 2 | | | AC[a] and research team |
|---|---|---|---|---|---|---|---|
| | Basket | Trash | Adding | Basket | Trash | Adding | Decision |
| Being guided in the help-seeking process | ✓ | — | — | Modified to: Being guided in identifying needs and in the process of discreetly seeking help | — | — | Kept |
| Being guided in identifying needs | — | ✓ | — | | — | — | Replaced by: Recognizing the needs |
| Recognizing the problem | Modified to Recognizing the needs | — | — | — | ✓ | — | |
| Being reassured about resources | ✓ | — | — | — | ✓ | — | Kept |
| Asking questions | Modified to: Having access to | — | — | — | ✓ | — | Kept with the original wording |

| Initial needs | Co-design 1 | | | Co-design 2 | | | AC[a] and research team |
|---|---|---|---|---|---|---|---|
| | Basket | Trash | Adding | Basket | Trash | Adding | Decision |
| | references that answer questions | | | | | | |
| Being able to keep and retrieve information easily | ✔ | — | — | ✔ | — | — | Kept |
| Receiving information regularly | ✔ | — | — | Modified to: Receiving regular information on what is offered by organizations across the territory (services, | — | — | Kept with the original wording |

| Initial needs | Co-design 1 | | | Co-design 2 | | | AC[a] and research team |
|---|---|---|---|---|---|---|---|
| | Basket | Trash | Adding | Basket | Trash | Adding | Decision |
| | | | | resources, and training) | | | |
| Recognizing themselves as caregivers | ✓ | — | — | ✓ | — | — | Kept |
| Feeling less guilty about using services | Modified to: Being comfortable using the services | — | — | Modified to: Being comfortable using the services | — | — | Kept with proposed modification: Being comfortable using the services |
| Finding resources | ✓ | — | — | — | ✓ | — | Rejected because: Comes with the tool |

| Initial needs | Co-design 1 | | | Co-design 2 | | | AC[a] and research team |
|---|---|---|---|---|---|---|---|
| | Basket | Trash | Adding | Basket | Trash | Adding | Decision |
| Having coordinated services | — | ✓ | | — | ✓ | — | Rejected because: The tool has no control over this |
| | — | — | Being informed about staff stability | — | — | — | Rejected |
| — | — | — | — | — | — | Being able to add training workshops, resources, and activities | Kept |

[a]AC: advisory committee.
